# Prevention of suicidal behavior in older people: A systematic review of reviews

**DOI:** 10.1371/journal.pone.0262889

**Published:** 2022-01-25

**Authors:** Lucie Laflamme, Marjan Vaez, Karima Lundin, Mathilde Sengoelge

**Affiliations:** 1 Department of Global Public Health, Karolinska Institutet, Stockholm, Sweden; 2 Department of Clinical Neuroscience, Karolinska Institutet, Division of Insurance Medicine, Stockholm, Sweden; University of Maryland School of Medicine, UNITED STATES

## Abstract

Older people have the highest rates of suicide, yet the evidence base on effective suicide preventions in late-life is limited. This systematic review of reviews aims to synthesize data from existing reviews on the prevention and/or reduction of suicide behavior in late-life and evidence for effectiveness of interventions. A systematic database search was conducted in eight electronic databases from inception to 4/2020 for reviews targeting interventions among adults ≥ 60 to prevent and/or reduce suicide, suicide attempt, self-harm and suicidal ideation. Four high quality reviews were included and interventions categorized as pharmacological (antidepressant use: 239 RCTs, seven observational studies) and behavioral (physical activity: three observational studies, and multifaceted primary-care-based collaborative care for depression screening and management: four RCTs). The 2009 antidepressant use review found significant risk reduction for suicide attempt/self-harm (OR = 0.06, 95% CI 0.01–0.58) and suicide ideation (OR = 0.39, 95% CI 0.18–0.78) versus placebo. The 2015 review found an increased risk of attempts with antidepressants versus no treatment (RR = 1.18, 95% CI 1.10–1.27) and no statistically significant change in suicides versus no treatment (RR = 1.06, 95% CI 0.68–1.66) or ideation versus placebo (OR = 0.52, 95% CI 0.14–1.94). Protective effects were found for physical activity on ideation in 2 out of 3 studies when comparing active versus inactive older people. Collaborative care demonstrated significantly less attempts/ideation (OR = 0.80, 95% CI 0.68–0.94) in intervention group versus usual care. The results of this review of reviews find the evidence inconclusive towards use of antidepressants for the prevention of suicidal behavior in older people, thus monitoring is required prior to start, dosage change or cessation of antidepressants. Evidence to date supports physical activity and collaborative management for reduction of suicide ideation, but additional trials are required for a meta-analysis. To build on these findings, continued high-quality research is warranted to evaluate the effectiveness of interventions in late life.

## Introduction

Older persons have the highest mortality rate due to suicide in almost all regions of the world [1]. Men’s prevalence is higher than that of women, in part because they tend to use more lethal suicide methods (e.g., hanging, jumping, sharp objects) [2]. Certain risk factors are comparable to those observed in other age segments of the population; these include psychiatric conditions, feelings of hopelessness, depression or prior suicide attempts [3]. Yet older people face particular age-related challenges such as poorer physical and mental health, pain, cognitive deficits, co-morbid medical conditions that impair function or life expectancy, increased frailty and limited social connectedness [4]. An additional important factor is the presence of somatic comorbidities that may afflict older people, such as neurological diseases, pain and oncological diseases, which have been found to occur more frequently in older people exhibiting suicidal behaviour [5]. Neurological diseases in particular cause biological impairments as well as feelings of severe hopelessness, both linked to increased vulnerability to suicidal behavior [6]. It has also been shown that older people with previous suicide attempts or who die by suicide are more likely to have a plan and are more determined than younger adults, resulting in higher lethality rates [7]. They are also less likely to disclose emotional distress, thus there also exists the problem of underreporting of the burden of suicide in the aged [8].

Suicidal behavior in older people is a result of numerous interactions that are dynamically changing as people age. This makes its detection and the development of effective preventive interventions to tackle this behavior challenging. Although no model to date is specific to late life suicide, a number of models have been developed to advance understanding of the interplay of the biological, clinical, psychological, social, cultural risk and protective factors involved in suicidal behavior. Examples include the Interpersonal Theory of Suicide that emphasizes the role of acquired capability and the simultaneous presence of thwarted belongingness and perceived burdensomeness that transact with the interpersonal environment [9] and the Stress-Diathesis model that posits suicide is the result of an interaction between state-dependent (environmental) stressors and a trait-like diathesis or susceptibility to suicidal behavior [10]. These models have been applied in the suicide field for a social-ecological approach to prevention and link to efforts to identify the social determinants of mental health using a life course approach [11]. Various reviews have been conducted on suicide prevention of interventions appropriate for all age groups and effective diagnosis and treatment of depression is most often cited as a preventive intervention because of the close association between affective illness and suicidal behavior in older people [12–14]. Yet controversy still remains as to the potential risks involved and that affective illnesses may have low detection rates [14]. A systematic review completed in 2011 by Lapierre and colleagues identified the numerous interventions utilized for prevention of suicidal behavior in older people: primary care interventions, community-based outreach, telephone counselling, pharmacotherapy and cognitive-behavioral therapy or psychotherapy [15]. This heterogeneity in interventions has prevented the potential for a meta-analysis. The review found that the majority of studies used only the presence or absence of completed suicide as the outcome and most of the interventions targeted the reduction of risk factors [15]. While some of the interventions identified are applicable to all ages (e.g. pharmacotherapy), a few are unique to late life and differ from other age groups. For example, older adults are less likely to utilize mental health services compared to younger adults as this aged population tend to present to primary care services [16].

There is a wide range of interventions and settings utilized to reach and intervene with older adults at risk for suicide. The many individual studies and several reviews existing to date have employed different definitions of suicide, included certain forms of interventions and excluded others (e.g., multilevel or behavioural or pharmaceutical) and used narrow outcome measures, e.g., only death by suicide. As a result, there is a lack of clarity about what the evidence at hand shows and what interventions are effective from a cross-disciplinary perspective. A systematic review of original articles performed by the authors identified only four new studies that were not already included in an existing review. Noting the number of systematic reviews performed to date, a systematic review of reviews was selected as a tool to enable the findings of reviews to be synthesized, compared and contrasted. This allows for providing a single comprehensive overview of the published literature that includes multiple intervention types and a variety of suicide behavior outcomes.

The objectives of this systematic review of reviews were two-fold: 1) to synthesize data from existing reviews on the prevention and/or reduction of suicide behavior in older adults, including the characteristics of relevant reviews, the definition of suicide behavior used, the types of interventions included and their objectives, outcomes and effects; and 2) to analyze the evidence for effectiveness. The results are to contribute to identification of knowledge gaps on what interventions are effective to guide future research.

## Materials and methods

### Search strategy

A systematic search strategy was developed following the Systematic Reviews and Meta-Analyses checklist (S1 Table) and applied to the following eight electronic databases: Medline, Epub Ahead of Print, In-Process & Other Non-Indexed Citations, Ovid MEDLINE(R) Daily and Ovid MEDLINE(R) (Ovid); Psycinfo (Ovid); Embase.com; Web of Science Core Collection; CINAHL (Ebsco); Cochrane Library (Wiley); SveMed+ and Google Scholar. The original search period was January 2000 to April 2017 and a second search was repeated in April 2020 using the same search protocol. As an additional method of review identification, we checked the reference lists of selected articles to detect any missed studies using the snowballing technique. Further details on the full electronic search strategy is included as a S2 Table. All records were imported into the electronic reference management software, EndNote (version X8). To identify reviews, the following keywords were searched for in the EndNote library: review OR overview OR meta-analysis (each search separate).

### Eligibility criteria

The PICO components (population, intervention, comparator and outcome) and exclusion criteria are depicted in Table 1. We selected the age group ≥60 years in order to maximize the potential inclusion of reviews. Furthermore, publications included any type of review (scoping, systematic, systematic review including a meta-analysis) published in the peer-reviewed literature from January 1, 2000 to April 1, 2020. One or more suicidal behavior outcome was included in order to capture all older adults who would benefit from prevention measures in the pathway ranging from suicidal ideation to completed suicide. Reviews were excluded if a more recent review existed that included all of the studies from the earlier review. No language restrictions were applied.

**Table 1:** Inclusion and exclusion criteria.

PICO	Inclusion	Exclusion
Population	Adults ≥ 60 years old as target population or subgroup	Population without adults ≥ 60 years old
Intervention	Any type of intervention to prevent or reduce suicidal behavior	Intervention without a suicide prevention component; physician assisted suicide, euthanasia
Comparator	Individuals or groups who did not receive the target intervention	No comparator or control
Outcome measures	Any type of outcome on suicidal behavior defined as suicide, suicide attempt, self-harm, suicidal ideation	No suicidal behavior outcome data

### Data extraction, analysis and quality assessment

Titles and abstracts of retrieved records were screened to identify reviews that met the inclusion criteria. At the first stage studies with relevant titles were selected for second screening by two reviewers evaluating independently of one another (MS, KL). At the second stage, only those abstracts satisfying inclusion criteria were retained for full-text review. The reviewers also examined the reference lists of all included data to identify other potentially eligible reviews. No disagreements were found within the review team regarding inclusion. One reviewer (MS) extracted data using a predetermined data extraction form based on the PRISMA checklist which was subsequently independently verified by the second reviewer (KL). No contact was needed with study authors to obtain or confirm data.

Interventions had to be described in sufficient detail to enable classification as suicide prevention. The outcome measures were to include the impact of interventions on suicide, suicide attempts, self-harm without suicide intent and/or suicidal ideation. Two reviewers (MS, KL) independently completed a methodological quality assessment of the included reviews. The AMSTAR2 tool was used as it is especially designed to assess the quality of systematic reviews and meta-analyses based on 16 quality criteria and seven critical domains [17]. Reviews are rated as high if they have zero or one non-critical domain; moderate if more than one non-critical domain, and low if one critical flaw with or without non-critical weaknesses. A review that scored an overall rating of low was removed from the analysis. The reviewers were consistent in their decision on inclusion. In order to analyze the reviews systematically, the following data were extracted: description of the patient or participant group/sample studied, nature of the intervention covered in the review, type of outcomes investigated, setting (clinical or population-based) type of review, and methodological elements such as the number of included studies, search time frame and major findings. The included reviews were then synthesized according to the Synthesizing Without Meta-analysis (SWiM) reporting guideline to promote reporting for reviews of interventions that use alternative synthesis methods [18]. We adapted the guideline such that findings of ‘studies’ were noted as findings of ‘reviews’. The nine-item SWiM checklist was used to report how the reviews were grouped, the metric used for the synthesis, the synthesis method including how data were presented and limitations of the synthesis.

## Results and discussion

**Fig 1:**
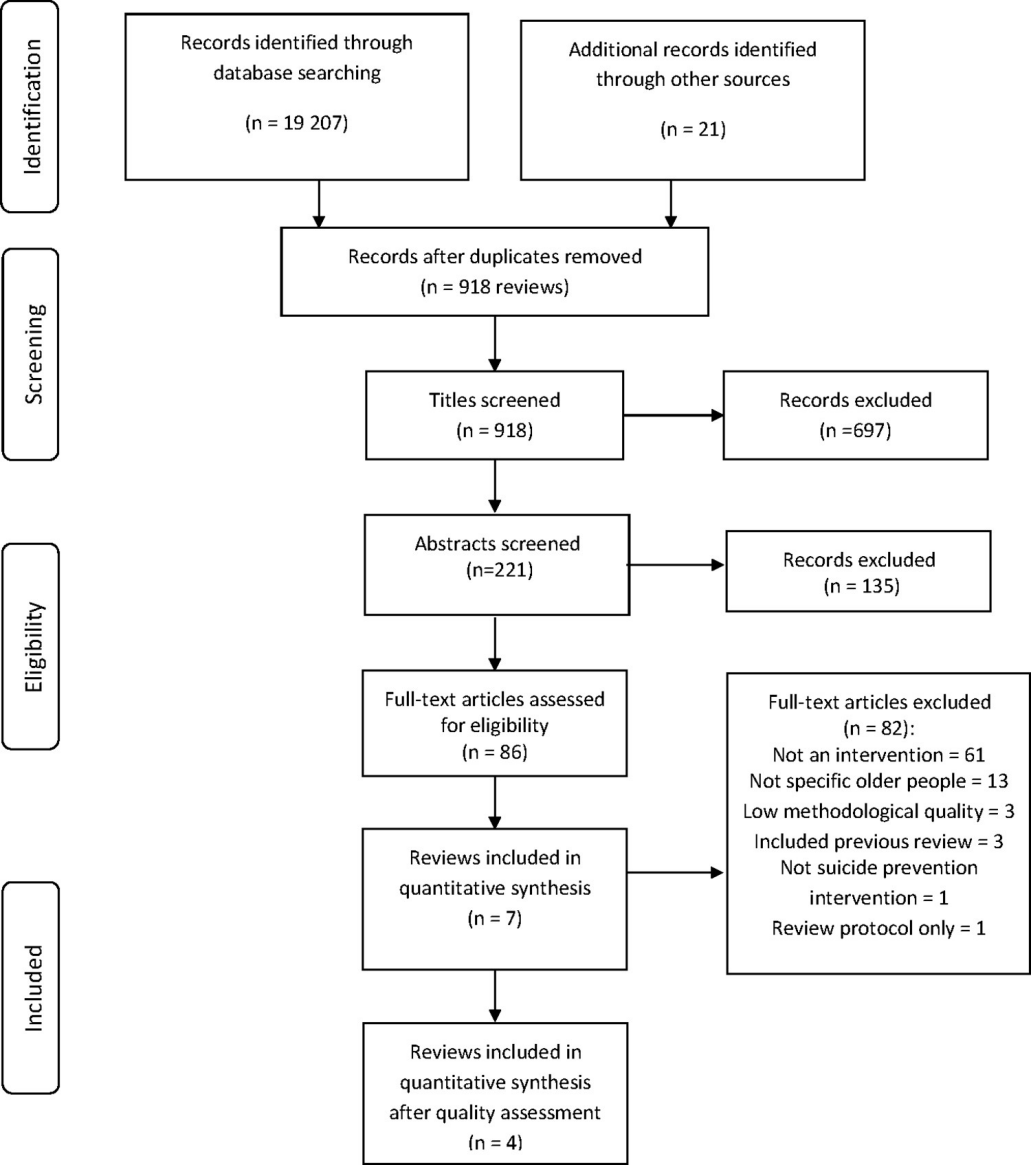
Flow diagram of the selection and review process.

The search generated 731 records identified as reviews. Screening of titles resulted in 221 records. Further screening of abstract and full-text resulted in a total of seven reviews evaluating interventions to reduce or prevent suicidal behavior in older people. The flow chart depicting the search strategy is presented in the Preferred Reporting Items for Systematic Reviews and Meta-Analyses (PRISMA) Flow Diagram (Fig 1).

The main reasons for exclusion of 82 records based on full-text review were that the population was not specific to older people (i.e. all ages, data aggregated) and focus on risk factors for suicide, not interventions. The seven reviews were assessed for methodological quality. Three reviews on interventions that influence help-seeking and psychiatric health care utilization among individuals with suicidal behavior [19], community interventions for late-life suicide [20] and multifaceted, selective and targeted interventions for late-life suicide prevention [21] scored an overall low level of evidence (AMSTAR2 <11) and were excluded. The review by Lapierre and colleagues [15] from 2011 was excluded and not assessed for quality as the studies in this review were included in the Okolie et al. review that met the inclusion criteria [22]. Thus, a total of four reviews were included. The reviews were categorized by intervention type, pharmacological (n = 2) and behavioral (n = 2) and the direction of effect was analyzed. Next, vote counting of the effect direction was utilized to summarize the results; see the S3 Table for full details of the SWiM reporting guideline.

### Characteristics of included reviews

Two reviews were systematic [22, 23] and two included a systematic review and meta-analysis [24, 25]. The reviews presented findings from randomized control trials as well as observational, prospective and cross-sectional studies representing a range of high-income countries: Canada, Denmark, Europe, Japan, South Korea, the Netherlands, United Kingdom (UK), USA. Only one study on antidepressants by Barak et al. [26] was present in more than one review [22–24].

Two reviews were exclusively on adults ≥ 60 years [22, 24] and the other two had a broader age scope (all adults and all ages) but included older adults and provided results in a disaggregated manner [23, 25]. Table 2 summarizes the characteristics of the four reviews categorized by type of intervention, pharmacological and behavioral. The table includes information on the type of review, description of the intervention, number of databases searched, search time frame, languages searched, countries, population under study, type of outcome measures covered, and number of studies included.

**Table 2:** Characteristics of four included reviews.

Author, year	Type of review	Intervention	# Databases/ Time frame/ Languages/ Countries	Population	Outcome	Number of studies
**Pharmacological**						
**Antidepressant use**						
O’Connor 2009 [23]	Systematic review	Drug: Antidepressants including selective serotonin reuptake inhibitors and other second-generation drugs compared to placebo	5/ 1998–2007/ English only/ The Netherlands, Non-North American and North American, UK, USA	All adults with depression	Suicide	7 reviews/meta-analyses: total of 3 observational studies with 2 stratifying results on older adults ≥ 65 and experimental studies, approx. 233 RCTs
					Suicide attempts and serious self-harm	
					Suicide ideation	
		Dose: clinically effective dose recommended by manufacturer				
		Treatment time: less than 8 weeks and greater than 8 weeks (mean time of treatment varied, 24 weeks to 3 years)				
KoKoAung 2015 [24]	Systematic review and meta-analysis	Drug: Selective serotonin reuptake inhibitors medication compared to placebo, no treatment or other antidepressant	15/ Not specified/ English only// Canada, Europe (11 countries), France, Germany, Israel, UK, USA	Ages ≥ 60 with depression	Suicide	5 observational studies, 6 RCTs
					Suicide attempts	
					Suicide ideation	
		Dose: clinically effective dose recommended by manufacturer				
		Treatment time: min. 4 weeks to 11 years				
**Behavioral**						
**Physical activity**						
Vancampfort 2018 [25]	Systematic review and meta-analysis	Any physical activity intervention that uses bodily movement produced by skeletal muscles and requires energy expenditure	7/ Inception to 5.2017/ No language restriction/ Australia, South Korea	All ages: 11 studies adolescents, 15 studies adults, 3 studies adults 65 +	Suicide ideation	29 total, 3 on older adults 65+ of which 2 cross-sectional, 1 prospective
**Multifaceted**						
Okolie 2017 [22]	Systematic review	1) Primary care (n = 4): collaborative care on depression screening and management programs	5/ Inception to 1.4.2016/ English only/ Australia, France, Germany, Hong Kong, Israel, Japan, USA	Ages 60+	Suicide Suicide attempt and ideation Suicide ideation	21 (RCTs, case- control, cohort, quasi- experimental, before and after)
		2) Clinical-based (n = 6): pharmacotherapy (n = 3) and psychotherapy (n = 3)				
		3) Community-based (n = 11): multilevel programs (n = 8) and telephone counselling (n = 3)				

Three outs of the four reviews restricted their search in English and a range of five to 15 databases were searched among all four reviews.

#### Study quality

The detailed quality assessment of the seven reviews having met the initial inclusion criteria are summarized in the S4 Table. The quality rating presented in Table 3 indicates that the four included reviews had a high level of evidence and did not have any critical weaknesses.

**Table 3:** Synthesis of results.

Intervention	Outcome	Effect		Quality
Pharmacological				
Antidepressant use				
O’Connor, 2009 [23]	**Suicide**	No evidence		High
	Experimental (233 RCTs): no evidence as no suicides documented in treatment with antidepressant drug exposure compared to placebo			
	**Suicide attempt, serious self-harm**	↓		
	Experimental (233 RCTs): significantly lower risk in second-generation antidepressant drug exposure compared to placebo (OR = 0.06, 95% CI 0.01–0.58)	↓		
	**Suicide ideation**			
	Experimental (233 RCTs): significantly lower risk in second-generation antidepressant drug exposure compared to placebo (OR = 0.39, 95% CI 0.18–0.78)			
KoKoAung, 2015 [24]	**Suicide**	−		High
	Observational (2 studies, n = 100 343):			
	no statistically significant change in risk in seven to 11-year use of selective serotonin reuptake inhibitors medication compared to no treatment (RR = 1.06, 95% CI 0.68–1.66)	↑		
	**Suicide attempt**			
	Observational (3 studies, n = 132 306): 18% increased risk with 2 to 11-year selective serotonin reuptake inhibitors medication compared to no treatment (RR = 1.18, 95% CI 1.10–1.27)			
		−		
	Experimental (4 RCTs, n = 592): no statistically significant difference in risk with selective serotonin reuptake inhibitors medication compared to other antidepressants (OR = 1.00, 95% CI 0.14–7.10)	−		
	**Suicide ideation**			
	Experimental (2 RCTs, n = 1 281): no statistically significant difference in risk in selective serotonin reuptake inhibitors medication compared to placebo (OR = 0.52, 95% CI 0.14–1.94)			
**Behavioral**				
Physical activity				
Vancampfort, 2018 [25]	**Suicide ideation** (n = 50 745): 2 out of 3 studies (67%) demonstrated higher physical activity levels were significantly associated with lower suicide ideation when comparing active versus inactive	↓		High
	Study 1: Significant correlation (r = −0.102, P<0.01)			
	Study 2: Significantly higher in inactive vs. active (OR = 3.17, 95% CI 2.18–4.60)			
	Study 3: No significant correlation when physically active alone (r = −0.12) or with others (r = −0.13)			
Primary care-based collaborative screening and management of depression				
Okolie, 2017 [22]	**Suicide attempt (**1 cluster RCT, N = 599): 2 patients intervention arm, three patients usual care arm	↓		High

		↓		
	**Self-harm (composite measure of suicide attempt and ideation)** (1 cluster RCT, n = 21 762 patients): Significantly less over 24 months in intervention group vs. control group (OR = 0.80, 95% CI 0.68–0.94)	↓		
		↓		
	**Suicidal ideation** (4 cluster RCTs: n = 16 708; n = 1,801; n = 21 762; n = 599):			
		−		
	Short-term—significantly lower in primary collaborative care vs. usual care			
	At 4 months: intervention 12.9% vs. usual care 3.0%, p = 0.01 and 12.8% vs. usual care 3.0%, p = 0.02; no OR values provided			
	At 6 months: intervention 7.5% vs. usual care 12.1%, p = 0.001; OR 0.54, 95% CI 0.37–0.78			
	At 8 months: intervention 12.2% vs. usual care 1.5%, p = 0.003; no OR values provided			
	Long-term, 24 months—significantly lower in one cluster RCT but not the other one			
	Cluster RCT: 10.1% vs. 13.9%; OR 0.65, 95% CI 0.46–0.91, p = 0.01			
	Cluster RCT: intervention 18.3% vs. usual care: 8.3%; p = 0.12, no OR values provided			

RCT: randomized control trial, OR: odds ratio, CI: confidence interval, RR: relative risk.

### I. Pharmacological interventions

Two reviews investigated the association between suicide and antidepressant use treatment, O’Connor et al. [23] in 2009 followed by KoKoAung et al. [24] in 2015. Both included fatal and non-fatal suicidal behavior outcomes. The O’Connor et al. review investigated the effect of second-generation antidepressants, particularly selective serotonin reuptake inhibitors (SSRIs), on suicidal behavior in all ages and two studies reported stratified results on older adults based on approximately 233 RCTs. The more recent KoKoAung [24] review examined the effect of SSRIs specifically compared to other antidepressant use or placebo in RCTs and observational studies (population cohort and retrospective case control studies). Two RCTs compared SSRIs versus placebo on suicidal ideation and four RCTs and three observational studies compared SSRIs versus other antidepressants on suicide attempt. In addition, two observational studies provided data on completed suicide and three on suicide attempt. There were no data available on suicidal ideation from the observational studies included in the review.

### II. Behavioral interventions

#### Physical activity

The review by Vancampfort et al. [25] investigated physical activity in clinical and non-clinical populations comparing “active” to “inactive” as defined by the studies as well as meeting physical activity guidelines compared to not meeting these guidelines, defined as 150 minutes per week of at least moderate or 75 min per week of vigorous physical activity. Three of the studies (two from South Korea, one from Australia) focused specifically on older people; one a longitudinal study and the other two cross-sectional.

#### Multifaceted behavioral interventions

The review by Okolie et al. [22] encompassed multifaceted interventions in three settings: a) primary care for depression screening and management, b) clinical-based (pharmacotherapy and psychotherapy) and c) community-based. The majority of the interventions addressed risk predictors such as depression and in which manner the interventions varied. They included primary care-based depression screening and management programs in which training was provided to primary care physicians on assessment and management of both depression and suicidal behavior; pharmacotherapy with antidepressants; psychotherapy in the form of Problem Solving Therapy, Problem Adaptation or Supportive Therapy; telephone counseling for vulnerable older adults via a 24-hour Friendship Line or geriatric outreach via schedule telephone calls; and community-based programs incorporating education, gatekeeper training, depression screening, mental health/health education workshops; participating in social, voluntary and recreational activities; and exercising together.

### Intervention and its effects

Table 3 provides the key findings for each review by intervention and suicidal behavior outcome: suicide; suicide attempt/serious self-harm; suicidal ideation.

#### Suicide

The 2009 review on antidepressant use included SSRIs and other second generation drugs was not able to assess the association with suicide as no suicide deaths were reported in the approximately 233 RCTs. In contrast the 2015 review on antidepressant use focused exclusively on long term use (two to 11 years) of SSRIs found no statistically significant decreased risk for suicide (RR = 1.06, 95% CI 0.68–1.66) based on two observational studies (n = 100,343).

#### Suicide attempts, self-harm, suicide ideation

The experimental studies reviewed by O’Connor et al. revealed a significantly lower OR with antidepressants compared to placebo for both suicide attempt and serious self-harm (OR = 0.06, 95% CI 0.01–0.58) and suicide ideation (OR = 0.39, 95% CI 0.18–0.78). In the studies included by KoKoAung et al. concerning long-term SSRIs compared to no treatment, experimental studies found no lower odds of suicide attempts (OR = 1.00, 95% CI 0.14–7.10; n = 592) or suicide ideation (OR = 0.52, 95% CI 0.14–1.94; n = 1 821). In addition, in the latter review observational studies demonstrated that those with long-term SSRIs had an increased risk of suicide attempts compared to those without treatment (RR = 1.18, 95% CI 1.10–1.27).

For its part, the review on physical activity was based mainly on cross-sectional studies comparing “active” versus “inactive” persons (as defined by each study) and those who met the recommended international physical activity guidelines (150 min per week of at least moderate or 75 min per week of vigorous intensity physical activity) versus those who did not. Older “active” persons had significantly lower odds of suicide ideation compared to “inactive” ones in two out of the three studies (67%; n = 50,745).

The Okolie et al. review on multifaceted interventions found that of the three types of interventions studied, only primary-based collaborative care was of sufficient methodological quality. This care refers to case managers and physicians working together on identifying and managing depression and risk of suicide, demonstrating a reduction of self-harm (attempts and ideation) and suicide ideation at four, six and eight months. Results at 24 months were mixed with one cluster RCT reporting no significant difference (p = 0.12) and two other cluster RCTs showing significantly lower levels of ideation (p = 0.01; OR 0.80, 95% CI 0.68–0.94).

This systematic review of reviews synthesized data from existing reviews targeting the prevention or reduction of suicidal behavior in older people. The four included reviews were assessed as being of high methodological quality, but not all included studies were sufficiently powered to study suicide or did not include this outcome due to its low incidence. This may be due to several reasons, such as the low base rate of suicidal behavior in older (2–4 attempts for each suicide death) compared to younger ages (200 attempts for each suicide in some adolescent and young adult samples)[27]; the frailty of older adults resulting in high dropout rates in studies and the difficulty in inclusion of older adults at risk for suicide in outcomes research due to their isolation in society, comorbidities and functional impairments [28]. Assessing multiple reviews enabled us to integrate data from a large number of studies and the small degree of overlap (one study) limited the risk of duplication of conclusions while ensuring the reviews were not overly selective.

Although antidepressant medication for treatment of depression in older people has been investigated over a long period of time in both experimental and observational study designs [13, 28], our review suggests that data from the 2015 review of SSRIs showed no evidence of a protective effect for suicide or suicidal ideation [24]. As SSRIs may also be prescribed for mood disorders other than depression [29, 30], there may be an indication bias. One other challenge when comparing studies on antidepressants is that the population included in RCT studies may include a population at higher risk. Thus, a matter of heterogeneity may be present. Also, the high dropout rate in the studies included as noted by KoKoAung et al. may have led to worsening of depression and therefore predispose older persons to increased risk of suicidal behavior [24]. Results from these types of studies examining outcomes for pharmacological treatment versus no treatment are difficult to interpret due to potential confounding by indication. Physicians may have been more likely to prescribe antidepressants for older persons with more severe depression, who in turn were also more likely to thus have suicidal behaviors. As the studies were observational there was no control or experimental group to control for this. As the sale of antidepressants is rising globally [31, 32], it is necessary to continue scrutinizing the data in order to optimize the use of antidepressants. Furthermore, other drugs used in mood disorders have shown some efficacy for prevention of suicidal behavior in adults, and thus warrant further study in older populations [33].

Investigating protective factors, the Vancampfort et al. review showed that physical activity compared to being inactive had protective effects on suicidal ideation in two out of three studies on older people. As this result was based mainly on cross-sectional data, additional prospective observational studies and controlled trials are needed to confirm the findings. Furthermore, as the majority of studies did not include a comprehensive physical activity assessment, data are lacking on what type and dose (i.e. length, frequency, duration) of physical activity would be optimal for older people. This information is crucial to guide prevention programs as it is not known to what extent older adults with serious physical illness and functional limitations (both associated with suicidal behavior [5]), may be physically capable of 75 minutes of rigorous exercise per week. Also, those with depression may not feel able to exercise regularly due to lack of energy and motivation. Nonetheless, intervention strategies for older people may target the importance of physical activity for prevention of suicidal ideation based on these preliminary findings. It is key that public health research focuses on the impact of general health promotion to optimize successful aging across the health continuum [34] and capture whether such an approach may synergistically reduce suicidal behavior [14].

The review encompassing multifaceted interventions aimed at addressing risk predictors for suicidal behavior and ideation found that collaborative management in primary care was associated with reduced self-harm (composite measure of suicide attempt and suicide ideation) and for reduced suicidal ideation [22]. This finding can be explained in that older people may often have physical and mental health symptoms or conditions that need to be addressed, which are optimally addressed and treated in a collaborative, holistic team approach. It is important to recognize that that the inclusion of self-harm with and without suicidal intent as well as suicidal ideation may be problematic as although they have overlapping behaviors, they display some distinct risk profiles [35]. In addition, some older adult victims may not disclose any level of ideation [36] and ideators may never transition to suicidal behavior [37]. Yet, a history of non-suicidal self-harm and of suicidal ideation were shown by meta-analysis to confer a later risk of suicidal thoughts and behavior ranging from ideation to attempts and death [38]. Thus, further research focused on improving understanding of the determination of intent and interpretation of suicidal behavior in this age group would be informative. Furthermore, no systematic attempt was made in this review to identify possible synergies between interventions, although there is evidence that combinations of preventive interventions at several levels may have potential synergistic effects, e.g. training of general practitioners to address both depression and social isolation in older patients [39]. Previous research highlights the provision of general practitioner services due to reaching out to primary care prior to suicidal behavior and that services are accessible and integrated within other care elements to be effective in management and prevention [40].

In all four of the reviews included, the sustainability of the interventions and evidence of long-term effects beyond 24 months is unknown at this time. Longitudinal, large scale studies are required to assess this, and we identified only one multicenter study that demonstrated a reduced risk of repeated self-harm for persons of all ages combined receiving a psychosocial intervention in the short (one to five years) and long-term (10 to 20 years) compared to those receiving no intervention, as well as a reduced risk of death by suicide in the long-term; but the results were mixed when focusing on older people 50 years of age and older [41]. As the reviews identified in this study were few in number, it was not possible to stratify by gender.

### Strengths and limitations

This is to our knowledge the first systematic review of reviews to synthesize data from existing reviews on the prevention or reduction of suicidal behavior in older people. We included wide-ranging databases employed to identify documents of relevance and a systematic approach to screening, reviewing, assessing, and synthesizing in line with standard guidelines. Furthermore, we reported on fatal and non-fatal suicide outcomes. Yet several limitations exist. Firstly, there is limited information in the data presented regarding the frequency and duration of the interventions that were administered, in particular the behavioral ones. Secondly, we were unable to generate effect sizes because of the heterogeneity of the interventions or lack of meta-analyses in the original reviews. Thirdly, we limited the evidence to studies identified in previous systematic reviews and therefore do not capture interventions taking place that have not yet been the subject of a systematic review. For example, sedatives and hypnotics have been found to be associated with increased risk for suicide in adults ≥65 year in a case control study, even after adjustment for affective and anxiety disorders [42].

#### Directions for future research

This systematic review of reviews indicates there is a need for additional large-scale studies in order to build the evidence base of “what works” in suicide prevention among older people in order to intervene and alter the suicidal trajectories of older people [43]. This includes addressing the social and political determinants of suicidal behavior for a whole-of-society approach [44] given the widening gap in inequalities in suicide with higher rates among disadvantaged social groups and communities [45]. Our review identified no review addressing inequalities in late life suicide. This highlights the lack of a ‘fair opportunity of mental well-being,’ part of the World Health Organization Mental Health Action plan 2013–2020. To date, little data were found on interventions targeting high risk groups such as older men and older people who do not contact service providers, indicating the need for further investigation as to how to effectively reach these groups. Studies are also needed to examine the effect for the oldest old (≥75 years), as well as the effect of universal, population-based suicide interventions, e.g. suicide-specific funding for interventions tailored to older people. A number of interventions with promising results in other age groups or areas of mental health require testing in older people, such as cognitive behavioral therapy [46] or telemental health applications [47]. Furthermore, due to the recent meta-analysis finding of a significant effect size for multilevel interventions (different healthcare settings or domains and by different providers) on suicide and suicide attempts in persons of all ages, large scale studies are needed to determine if this is also of added value for older persons [48]. Lastly, as older people are an increasing population, the concomitant increase in life expectancy related to this demographic change means that aging-related neurological illnesses will continue to increase, such that older people may be at increased risk of suicidal behaviour. Interventions are needed to assess risk of suicide in older people newly diagnosed patients with neurological diseases [49].

## Conclusions

The results of this review find the evidence inconclusive towards the use of antidepressants for the prevention of suicidal behavior in older people and highlight that careful monitoring is required prior to the start, change in dose or cessation of antidepressants prescribed. Although evidence to date supports physical activity and primary care collaborative management for the reduction of suicide ideation, additional trials are required for evidence from a meta-analysis. To build on these findings, continued high-quality research is warranted to evaluate the effectiveness of interventions targeting suicidal thoughts and behavior in late life, particularly in older men and those who do not contact service providers.

## Supporting information

S1 Table PRISMA checklist. (PDF)

S2 Table Search strategy. (PDF)

S3 Table SWiM reporting guideline. (PDF)

S4 Table Quality assessment. (PDF)
