# Supplementary material for: Prevention of suicidal behavior in older people: A systematic review of reviews
Source: PLoS One. 2022 Jan 25;17(1):e0262889. doi: 10.1371/journal.pone.0262889 (PMC8789110; doi:10.1371/journal.pone.0262889)
Supplement: S2 Table — (PDF) [file pone.0262889.s002.pdf]

Supplemental data. Table 2. Protocol of systematic review of reviews

Date: April 2020

Original search November 2017 performed by: Klas Moberg, Karolinska Institutet Library

Databases:

1. Medline, Epub Ahead of Print, In-Process & Other Non-Indexed Citations, Ovid MEDLINE(R) Daily and Ovid MEDLINE(R) (Ovid)
2. Psycinfo (Ovid)
3. Embase (embase.com)
4. Web of Science Core Collection
5. Cinahl (Ebsco)
6. Cochrane (Wiley)
7. SveMed+
8. Google Scholar

Total number of hits:

- Before deduplication: 19 207
- After deduplication: 8011 then 2005 after deduplication against the old search result up to November 2017 (including 187 reviews, from 2015 to April 2020)

The MeSH-terms identified for searching Medline (OVID) were adapted in accordance to corresponding vocabularies in Psycinfo, Embase and Cinahl. Each search concept was also complemented with relevant free-text terms like: suicide, prevention, intervention, aged, elderly. Reviews 2015 to 2020 have been searched and sorted into the EndNote library.

1. Medline, Epub Ahead of Print, In-Process & Other Non-Indexed Citations, Ovid MEDLINE(R) Daily and Ovid MEDLINE(R)

Interface: Ovid<br><br>Date of Search: 2 April 2020<br><br>Number of hits: 3239<br><br>Comment: In Ovid, two or more words are automatically searched as phrases; i.e. no quotation marks are needed	Field labels:<br><br>• exp/ = exploded MeSH term<br>• / = non exploded MeSH term<br>• .ti,ab,kf. = title, abstract and author keywords<br>• adjx = adjacent within x words, regardless of order<br>• * = truncation of word for alternate endings

1. exp Suicide/
2. (suicid* or parasuicid*).ti,ab,kf.
3. or/1-2
4. Preventive Psychiatry/
5. Preventive Medicine/
6. Preventive Health Services/
7. Primary Prevention/
8. Health Policy/
9. Health Promotion/
10. Healthy People Programs/
11. Program Evaluation/
12. Program Development/
13. National Health Programs/
14. Mass Screening/
15. Public Health Surveillance/
16. exp Suicide/pc
17. (prevent* or program* or interven*).ti,ab,kf.
18. (suicid* adj5 (strateg* or reduc* or screening or surveillance or guideline* or action plan* or policy or policies)).ti,ab,kf.
19. or/4-18
20. exp Aged/
21. Aging/
22. Geriatric Psychiatry/
23. Geriatrics/
24. Health Services for the Aged/

25. (aged or aging or ageing or old or older or elderly or elders or geriatric* or psychogeriatric* or dementia or demented or community-dwelling or alzheimer* or late-life*).ti,ab,kf. not medline.st.

26. or/20-25

27. 3 and 19 and 26

28. remove duplicates from 27

29. limit 28 to yr="2000 -Current"

30. limit 29 to (comment or editorial or letter)

31. 29 not 30

2. Psycinfo

Interface: Ovid<br><br>Date of Search: 2 April 2020<br><br>Number of hits: 3282	Field labels:<br><br>• exp/ = exploded heading<br>• / = non-exploded heading<br>• ti,ab,id = title, abstract and author keywords<br>• adjx = adjacent within x words, regardless of order<br>• * = truncation of word for alternate endings

1. exp suicide/
2. attempted suicide/
3. suicidal ideation/
4. (suicid* or parasuicid*).ti,ab,id.
5. Suicidology/
6. or/1-5
7. suicide prevention/
8. suicide prevention centres/
9. prevention/
10. primary mental health prevention/
11. preventive medicine/
12. policy making/
13. exp health care policy/
14. government policy making/
15. health promotion/
16. exp mental health programs/
17. mental health program evaluation/
18. program evaluation/
19. program development/
20. screening/
21. health screening/
22. exp screening tests/
23. (prevent* or program* or interven*).ti,ab,id.
24. (suicid* adj5 (strateg* or reduc* or screening or surveillance or guideline* or action plan* or policy or policies)).ti,ab,id.
25. or/7-24
26. Aging/
27. geriatric psychiatry/
28. geropsychology/

29. geriatrics/

30. gerontology/

31. (aged or aging or ageing or old or older or elderly or elders or geriatric* or psychogeriatric* or dementia or demented or community-dwelling or alzheimer* or late-life*).ti,ab,id.

32. or/26-31

33. 6 and 25

34. limit 33 to ("380 aged " or "390 very old ")

35. 32 and 33 and 36. 34 or 35

37. limit 36 to yr="2000 -Current"

38. limit 37 to ("comment/reply" or editorial or letter) and 39. 37 not 38

3. Embase

<table>
<tr><td>Interface: embase.com

Date of Search: 2 April 2020

Number of hits: 3491

Comment: Emtree is the controlled vocabulary in Embase</td><td>Field labels:
/exp = exploded Emtree term
/de = non exploded Emtree term
ti,ab,kw = title, abstract and author keywords
NEAR/x = adjacent within x words, regardless of order
* = truncation of word for alternate endings</td></tr>
</table>

#33	#31 NOT #32
#32	[animals]/lim NOT [humans]/lim
#31	#29 NOT #30
#30	#29 AND ('conference abstract'/it OR 'editorial'/it OR 'letter'/it)
#29	#3 AND #19 AND #27 AND [2000-2020]/py
#28	#3 AND #19 AND #27
#27	#20 OR #21 OR #22 OR #23 OR #26
#26	#24 NOT #25
#25	(aged:ti,ab,kw OR aging:ti,ab,kw OR ageing:ti,ab,kw OR old:ti,ab,kw OR older:ti,ab,kw OR elderly:ti,ab,kw OR elders:ti,ab,kw OR geriatric*:ti,ab,kw OR psychogeriatric*:ti,ab,kw OR dementia:ti,ab,kw OR demented:ti,ab,kw OR 'community dwelling':ti,ab,kw OR alzheimer*:ti,ab,kw OR 'late life*':ti,ab,kw) AND [medline]/lim
#24	aged:ti,ab,kw OR aging:ti,ab,kw OR ageing:ti,ab,kw OR old:ti,ab,kw OR older:ti,ab,kw OR elderly:ti,ab,kw OR elders:ti,ab,kw OR geriatric*:ti,ab,kw OR psychogeriatric*:ti,ab,kw OR dementia:ti,ab,kw OR demented:ti,ab,kw OR 'community dwelling':ti,ab,kw OR alzheimer*:ti,ab,kw OR 'late life*':ti,ab,kw
#23	'gerontology'/de
#22	'geriatrics'/exp
#21	'aging'/de
#20	'aged'/exp
#19	#4 OR #5 OR #6 OR #7 OR #8 OR #9 OR #10 OR #11 OR #12 OR #13 OR #14 OR #15 OR #16 OR #17 OR #18
#18	(suicid* NEAR/5 (strateg* OR reduc* OR screening OR surveillance OR guideline* OR 'action plan*' OR policy OR policies)):ti,ab,kw
#17	prevent*:ti,ab,kw OR program*:ti,ab,kw OR interven*:ti,ab,kw
#16	'suicidal behaviour'/exp/dm_pc
#15	'screening test'/de
#14	'mass screening'/de

#13	'screening'/de
#12	'program development'/de
#11	'program evaluation'/exp
#10	'health promotion'/exp
#9	'health care policy'/de
#8	'prevention'/de
#7	'suicide prevention'/de
#6	'primary prevention'/de
#5	'preventive health service'/de
#4	'preventive medicine'/de
#3	#1 OR #2
#2	suicid*:ti,ab,kw OR parasuicid*:ti,ab,kw
#1	'suicidal behaviour'/exp

4. Web of Science Core Collection

<table>
<tr><td>Date of Search: 2 April 2020

Number of hits: 3293

Comments:</td><td>Field labels:
TS = Topic = title, abstract & keyword
NEAR/x = adjacent within x words</td></tr>
<tr><td colspan="2">TOPIC: (suicid* or parasuicid*)

AND

TOPIC: (prevent* or program* or interven*) OR TOPIC: (suicid* NEAR/5 (strateg* or reduc* or "screening" or "surveillance" or guideline* or "action plan*" or "policy" or "policies"))

AND

TOPIC: ("aged" or "aging" or "ageing" or "old" or "older" or "elderly" or "elders" or geriatric* or psychogeriatric* or "dementia" or "demented" or "community-dwelling" or alzheimer* or "late-life*")

Timespan=2000-2020

Refined by: [excluding] DOCUMENT TYPES: (MEETING ABSTRACT OR NEWS ITEM OR EDITORIAL MATERIAL OR LETTER)</td></tr>
</table>

5. Cinahl (Ebsco)

Date of Search: 2 April 2020<br><br>Number of hits: 1375<br><br>Comments:	Field labels:<br><br>• TI, AB = titel & abstract<br>• AU = authors<br>• MH = Cinahl Heading<br>• + = Exploded Cinahl Heading<br>• Nx = adjacent within x words

S1	(MH "Suicide+")
S2	TI (suicid* or parasuicid*) OR AB (suicid* or parasuicid*)
S3	S1 OR S2
S4	(MH "Preventive Health Care")
S5	(MH "Health Policy")
S6	(MH "Policy Making")
S7	(MH "National Health Programs")
S8	(MH "Program Development+")
S9	(MH "Health Screening")
S10	(MH "Suicide+/PC")
S11	TI (prevent* or program* or interven*) OR AB (prevent* or program* or interven*)
S12	TI (suicid* N5 (strateg* or reduc* or screening or surveillance or guideline* or "action plan*" or policy or policies)) OR AB (suicid* N5 (strateg* or reduc* or screening or surveillance or guideline* or "action plan*" or policy or policies))
S13	S4 OR S5 OR S6 OR S7 OR S8 OR S9 OR S10 OR S11 OR S12
S14	(MH "Aged+")
S15	(MH "Aging")
S16	(MH "Geriatric Psychiatry")
S17	(MH "Geriatrics")
S18	(MH "Health Services for the Aged")
S19	TI (aged or aging or ageing or old or older or elderly or elders or geriatric* or psychogeriatric* or dementia or demented or "community-dwelling" or alzheimer* or "late-life*") OR AB (aged or aging or ageing or old or older or elderly or elders or geriatric* or psychogeriatric* or dementia or demented or "community-dwelling" or alzheimer* or "late-life*")
S20	S14 OR S15 OR S16 OR S17 OR S18 OR S19

S21	S3 AND S13 AND S20
S22	S3 AND S13 AND S20 Limiters - Published Date: 20000101-20201231
S23	PT Commentary OR Editorial OR Letter
S24	S22 NOT S23

6. Cochrane (Wiley)

Date of Search: 2 April 2020<br><br>Number of hits: 240<br><br>Comments:	Field labels:<br><br>• ti,ab = titel & abstract<br>• au = authors<br>• near/x = adjacent within x words

#1 (suicid* or parasuicid*):ti,ab
#2 (prevent* or program* or interven*):ti,ab
#3 (suicid* near/5 (strateg* or reduc* or screening or surveillance or guideline* or "action plan*" or policy or policies)):ti,ab
#4 #2 or #3
#5 (aged or aging or ageing or old or older or elderly or elders or geriatric* or psychogeriatric* or dementia or demented or "community-dwelling" or alzheimer* or "late-life*"):ti,ab
#6 #1 and #4 and #5 Publication Year from 2000 to 2020

7. SveMed+

Date of Search: 2 April 2020<br><br>Number of hits: 42<br><br>Comments:	Field labels:<br><br>• exp = exploded MeSH term<br>• noexp = non exploded MeSH term

1	exp:"Suicide"
2	noexp:"Preventive Psychiatry"
3	noexp:"Preventive Medicine"
4	noexp:"Preventive Health Services"
5	noexp:"Primary Prevention"
6	noexp:"Health Policy"
7	noexp:"Health Promotion"
8	noexp:"Healthy People Programs"
9	noexp:"Program Evaluation"
10	noexp:"Program Development"
11	noexp:"National Health Programs"
12	noexp:"Mass Screening"
13	noexp:"Public Health Surveillance"
14	exp:"Suicide/prevention & control"
15	#2 OR #3 OR #4 OR #5 OR #6 OR #7 OR #8 OR #9 OR #10 OR #11 OR #12 OR #13 OR #14
16	exp:"Aged"
17	noexp:"Aging"
18	noexp:"Geriatric Psychiatry"
19	noexp:"Geriatrics"
20	noexp:"Health Services for the Aged"
21	#16 OR #17 OR #18 OR #19 OR #20
22	#1 AND #15 AND #21
23	year:[2000 TO 2020]
24	#22 AND #23

8. Google Scholar

Date of Search: 2 April 2020<br><br>Number of hits: 200<br><br>Comments: Google Scholar generally retrieves thousands of hits for every search, however only the first couple of hundreds tend to be of relevance.	Field labels:
"suicide|suicidal prevention" "program|intervention" "elderly|elders|aging|ageing|old|older|late life"<br><br>Custom range: 2000 – 2020	

Supplemental Table 1. Quality assessment scores based on AMSTAR2 for included reviews

Review	Criteria																Total "yes"
	1	2	3	4	5	6	7	8	9	10	11	12	13	14	15	16	
KoKoAung - antidepressant medication	Y	Y	Y	Y	Y	Y	Y	Y	Y	Y	Y	Y	Y	Y	Y	Y	16
O’Connor – antidepressant medication	Y	Y	Y	Y	Y	Y	Y	Y	Y	N	n/a	n/a	Y	Y	n/a	Y	12
Okolie – multifaceted interventions	Y	Y	Y	Y	Y	Y	Y	Y	Y	N	n/a	n/a	Y	Y	n/a	Y	12
Vancampfort – physical activity	Y	Y	Y	Y	Y	Y	Y	Y	Y	N	Y	Y	Y	Y	Y	N	14

N: no; Y: yes; n/a: not applicable if review did not include a meta-analysis; Total: maximum summative score of 16. Critical domains include criteria 2, 4, 7, 9, 11, 13, 15. Reviews are rated as high if they have zero or one non-critical domain; moderate if more than one non-critical domain, and low if one critical flaw with or without non-critical weaknesses.
