# Supplementary material for: Prevention of suicidal behavior in older people: A systematic review of reviews
Source: PLoS One. 2022 Jan 25;17(1):e0262889. doi: 10.1371/journal.pone.0262889 (PMC8789110; doi:10.1371/journal.pone.0262889)
Supplement: S4 Table — (PDF) [file pone.0262889.s004.pdf]

Supplemental Table 4. Quality assessment scores based on AMSTAR2 for review of reviews

First author/ Year/ Review topic	Criteria																Total "yes"
	**1**	**2**	**3**	**4**	**5**	**6**	**7**	**8**	**9**	**10**	**11**	**12**	**13**	**14**	**15**	**16**	
**KoKoAung** 2015 – antidepressant medication	Y	Y	Y	Y	Y	Y	Y	Y	Y	Y	Y	Y	Y	Y	Y	Y	16
**Vancampfort** 2018 – physical activity	Y	Y	Y	Y	Y	Y	Y	Y	Y	N	Y	Y	Y	Y	Y	N	14
**O'Connor** 2009 – antidepressant medication	Y	Y	Y	Y	Y	Y	Y	Y	Y	N	n/a	n/a	Y	Y	n/a	Y	12
**Okolie** 2017 – multifaceted Interventions	Y	Y	Y	Y	Y	Y	Y	Y	Y	N	n/a	n/a	Y	Y	n/a	Y	11
**Sais** 2013 – community interventions	Y	B	N	Y	Y	N	Y	N	N	N	n/a	n/a	N	Y	n/a	Y	6
**Hom** 2015 – help seeking, mental health service use	N	N	N	Y	N	N	N	Y	N	N	n/a	n/a	Y	N	n/a	Y	4
**Wyart** 2012 – prevention late life suicide	N	N	N	Y	N	N	N	Y	N	N	n/a	n/a	N	N	n/a	N	2

N: no; Y: yes; n/a: not applicable if review did not include a meta-analysis; Total: maximum summative score of 16
